# Elucidation of Exercise Conditions That Promote Motor Learning in Children With Autism Spectrum Disorder Who Have Motor Coordination Disorders: A Study Using Constant and Variable Practice

**DOI:** 10.7759/cureus.85987

**Published:** 2025-06-14

**Authors:** Soma Tsujishita, Daiki Nakashima, Kazunori Akizuki, Kosuke Takeuchi

**Affiliations:** 1 Department of Physical Therapy, Kobe International University, Kobe, JPN; 2 Department of Rehabilitation, Faculty of Health Science, Naragakuen University, Nara, JPN; 3 Department of Physical Therapy, Mejiro University, Saitama, JPN

**Keywords:** autism spectrum disorder, cooperative movement disorder, motor learning, practice conditions, social skill

## Abstract

Background and aim

Children with autism spectrum disorder (ASD) may experience coordination disorders, but the effects of different training conditions on motor learning in these children remain unclear. This study examined how constant versus variable practice impacts motor performance and learning in children with ASD and motor impairments.

Methods

Thirty-four children attending child development and daycare centers participated. Assessments included fine motor skills (Purdue Pegboard), gross motor skills (target-target task), visuospatial working memory (Corsi block tapping task), and the developmental disability questionnaire (Strengths and Difficulties Questionnaire). The primary outcome was the change in target-task performance before, during, and after practice, analyzed using repeated measures two-way ANOVA and Pearson’s correlation.

Results

No significant differences were observed for practice conditions or time effects alone, but significant interactions were found (F = 6.641, p = 0.015). Variable practice resulted in reduced pre- to post-test scores (p = 0.047), while constant practice showed stronger correlations between practice improvements and overall performance changes (p = 0.004, r = 0.666). Prosocial behavior was positively associated with performance improvements in the constant practice group (p = 0.018, r = 0.564). No significant correlations were found in the variable practice group.

Conclusions

Constant practice yielded greater motor learning improvements than variable practice. Additionally, prosocial behavior positively influenced motor learning in structured settings, highlighting the potential benefits of integrating motor and social skill interventions for children with ASD.

## Introduction

Recent reports indicate that approximately 6% of school-age children have motor coordination disorders [1]. Children with the condition are characterized by the inability to perform age-appropriate fine motor (handwriting and tying shoelaces) and gross motor (playing sports and getting dressed) activities [1]. This wide range of impairments not only affects the performance of daily tasks but also contributes to long-term adverse health effects secondary to decreased engagement in physical and social activities, low self-esteem, and increased risk of anxiety and depression [2,3]. In particular, it has been reported that 80-90% of children with autism spectrum disorder (ASD) have a co-occurring motor coordination disorder, which has become a problem [4].

Impairment of the internal model has been proposed as a neurological cause of coordination disorder [5,6]. Previous studies have reported that children with ASD associated with motor coordination disorder have dysfunctional sensory-motor integration in the internal model, resulting in a decreased ability to perform predictive motor control [5,6]. Therefore, it has been suggested that children with ASD associated with dyscoordinated movement disorders have difficulty with fine and gross motor control.

Although the internal model may be dysfunctional in children with motor coordination disorder, and their motor learning ability may be impaired, it has recently been reported that it is possible to promote motor learning even in children with motor coordination disorder [7,8]. van Cappellen-van Maldegem et al. conducted a three-week intervention study involving 24 children with developmental coordination disorder (DCD) aged six to 10 years [7]. Participants practiced a slinger ball throwing task while receiving either internal focus (body movement focused) or external focus (target focused) feedback. Results demonstrated that children with DCD showed improved throwing accuracy following practice, regardless of feedback type. However, external focus feedback led to superior retention of skills compared to internal focus. Crucially, the study revealed that children with DCD performed significantly worse under internal focus conditions than typically developing children, highlighting the detrimental effect of internal focus instructions for this population. de Carvalho et al. also examined the effects of the amount of practice on motor performance and motor learning in children with suspected motor coordination disorder [8]. The results revealed that the motor performance of children with suspected motor coordination disorder was lower than that of typically developing children in the early stages of practice, but motor learning occurred in the same manner as that of typically developing children, depending on the amount of practice. Thus, it is suggested that motor learning is possible even for children with motor coordination disorder. However, no previous studies have examined effective practice conditions to improve the efficiency of motor learning in children with ASD with motor coordination disorder.

A method based on schema theory, one of the motor learning theories, has been proposed as a practice method to promote motor learning in children and healthy adults [9]. Schema theory assumes that learners do not remember the sensations associated with each movement, but rather the rules (motor response schema) that exist between the generalized motor program of the execution of the movement and the results of the movement at that time. Therefore, it has been reported that practice with a great deal of variety (variety practice) is better suited for schema formation than practice with a fixed set of movements (constant practice) and that variety practice is superior in the performance of transition tasks not experienced in practice [9].

However, the difficulty children with ASD and motor coordination disorders experience in acquiring motor skills compared to typically developing children suggests that variable practice may yield inconsistent outcomes in this population. This finding aligns with Dick et al.’s study on Alzheimer’s disease (AD) patients, which demonstrated dysfunctional application of the variable practice theory in neurodegenerative populations [10]. They examined the effects of constant and variable practice conditions on a guessing task in 58 patients with AD and 58 healthy elderly subjects. The results showed that the healthy elderly subjects showed significant improvement before and after the task under all practice conditions, but the AD patients showed significant improvement only in the constant practice condition [10]. This result is thought to be because AD patients have impaired visuospatial working memory, which makes it difficult to process multiple-task information in parallel. This suggests that constant practice, which places less burden on visuospatial working memory, is more suitable for those who have difficulty forming motor schema, such as AD patients, than variable practice, which is believed to promote motor learning in children and healthy adults.

Thus, it has not been clarified what kind of practice methods are more effective for motor learning in children with ASD with motor coordination disorder, because effective practice methods for normal subjects are not always effective for those with disabilities. The characteristics of motor learning in children with ASD and the methods of practice that are most effective for them will help to reduce the negative impact of motor learning on their daily lives.

The purpose of this study was to investigate the effects of different practice conditions, such as constant practice and variable practice, on motor performance and its changes in children with ASD with motor coordination disorder and to examine practice methods that promote motor learning in children with ASD with motor coordination disorder.

## Materials and methods

Research design

This cross-sectional study was conducted from March 2023 to December 2023, targeting children attending after-school daycare service centers. The survey subjects were verbally informed of the purpose and content of the survey, that participation in the survey was voluntary, that they would not be disadvantaged if they did not respond to the questionnaire, that the survey could be terminated even after they consented to cooperate, and that they would not be disadvantaged in such cases. It was also explained that the respondents would not be identified because the data would be statistically processed. By signing the consent form, the respondents agreed to cooperate in this survey. This study was approved by the Ethics Committee of the Department of Physical Therapy, Faculty of Rehabilitation Studies, Kobe International University (approval no. G2023-177).

Subjects

**Figure 1:**
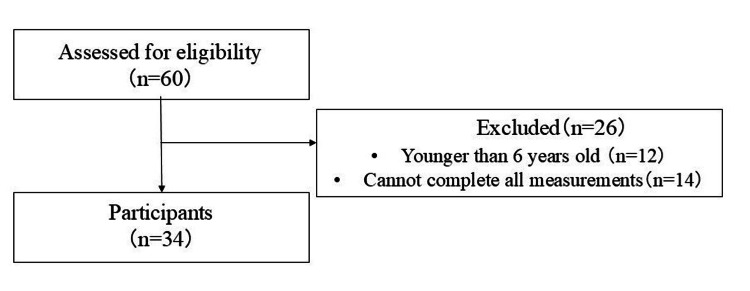
Flowchart of study participant selection

Participants comprised 60 children (aged six to 15 years) with physician-diagnosed ASD, recruited from a developmental support center in Osaka Prefecture. Eligibility requires teacher or guardian referrals due to movement difficulties warranting physical evaluation or therapy. Children under age six were excluded due to significant individual variations in neuromotor development that preclude reliable differentiation between DCD and typical developmental trajectories at this stage [1]. This age threshold aligns with established diagnostic protocols and methodological consensus in DCD research, where six to 15 years represents the optimal window for valid assessment of functional motor impairments [1]. Exclusion criteria were the inability to consent to participate in the study; being under six or 16 and older; diagnosis of cerebral palsy, muscular dystrophy, hemiplegia, degenerative disease, visual impairment, or intellectual disability; and missing measures. Data from 34 participants were included in the final statistical analysis (Figure 1).

The sample size was calculated using G*Power 3.1 software (Heinrich Heine University, Düsseldorf, Germany) with a power of 80%, alpha error of 0.05, and effect size of 0.25 (medium) for repeated measures two-way ANOVA. The sample size for repeated measures two-way ANOVA was calculated. The number of participants needed for this study was 34. To allow for the possibility of attrition, 60 participants were recruited.

Experimental tasks

**Figure 2:**
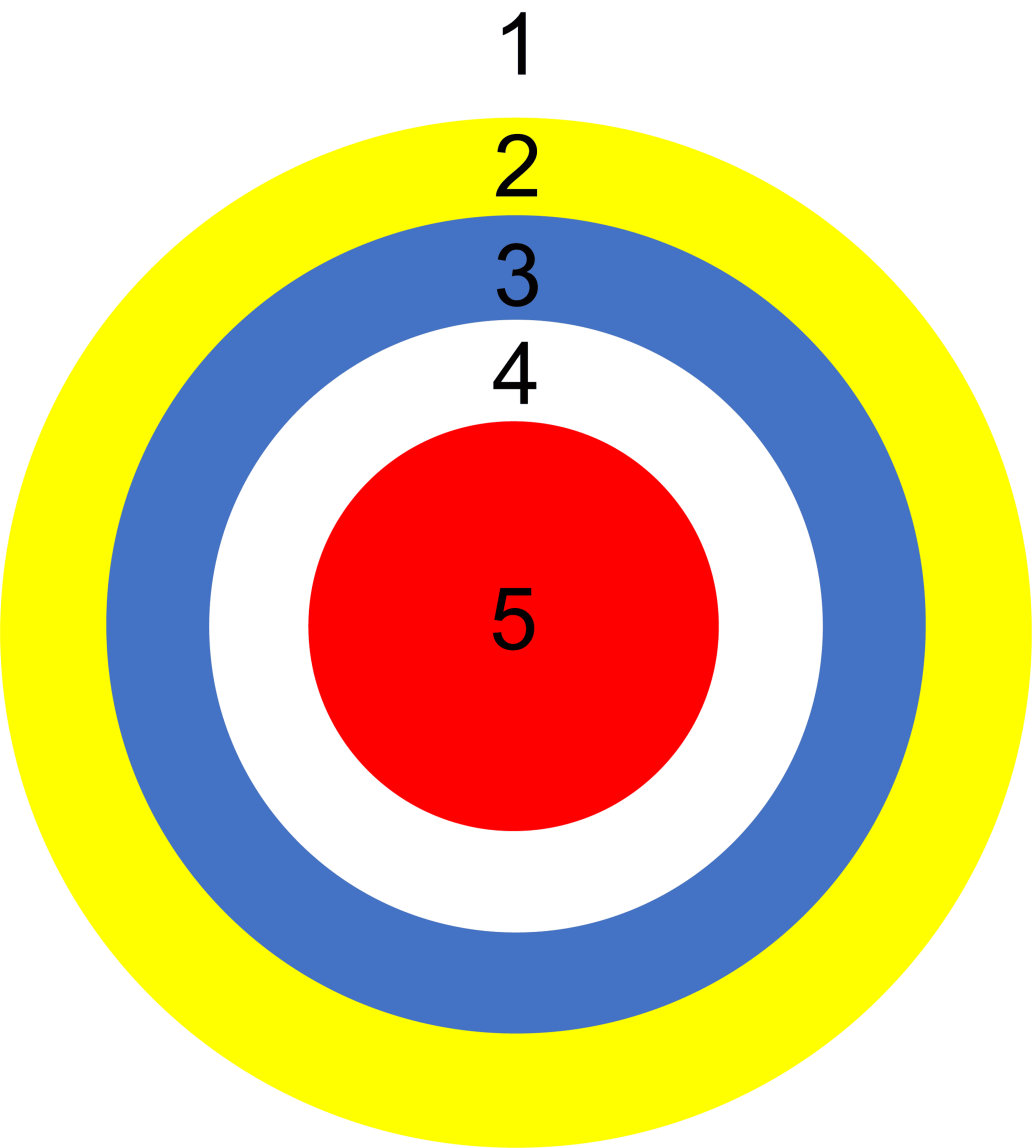
Gross motor test (targeting task)

The target-hitting task was performed as an experimental task for motor learning effects (Figure 2) [10]. The participants were asked to throw a 2 × 2-inch bean bag five times underhanded with their dominant hand toward a 36-inch “archery-type” target. Handedness was assessed through behavioral observation of three unilateral tasks: drawing, ball throwing, and spoon use. Participants demonstrating consistent right/left-hand use in ≥2 tasks were classified as right/left-handed, following the motor behavior assessment protocol validated in neurodegenerative populations [10]. The target was an 8-inch-diameter red circle surrounded by white, blue, and yellow 4-inch bands. Five points were awarded for hitting the center of the target, and lower points were awarded for hitting the outer circle, with a maximum score of 25 points. To adjust the difficulty level for each subject, the distance was set for each subject so that the average of five throws in advance was 1.5 points. As a precaution, we followed the implementation method of Dick et al. [10]. Specifically, the examiner explained and demonstrated the task to the study subjects at the beginning. If necessary, the examiner guided the participant's arm and asked him to perform the movement. The subject was asked to throw underhand with his dominant hand toward the target. If an invalid throw was made that did not meet these conditions, the participant was verbally corrected, and, if necessary, the task was performed again. In addition, examiners were trained to minimize verbal feedback by using standardized phrases. For a score of 5, they said, “Excellent, perfect, do it again!” For scores between 3 and 4, they said, “Very good, almost a success!” For scores between 1 and 2, they said, “Great!” while pointing to the center of the target and instructing, “Now throw it here!”

Measurement items

The items measured were motor coordination disorder (Purdue Pegboard), an experimental task (target-hitting task), visuospatial working memory (Corsi block tapping task, CBTT), and a questionnaire on developmental disabilities (Strengths and Difficulties Questionnaire, SDQ).

The Purdue Pegboard (A929-1 by Sakai Medical, Inc.) test is an instrument used to measure finger and hand dexterity [11]. The instrument consists of two rows of boards, each with 25 holes. The test requires the participant to place as many pins as possible in the 25 holes within 30 seconds, first with his preferred hand, then with his non-preferred hand, and finally with both hands. The total number of pins on each trial was used to score hand dexterity. The subjects were grouped to be homogeneous concerning the mean of their age.

**Figure 3:**
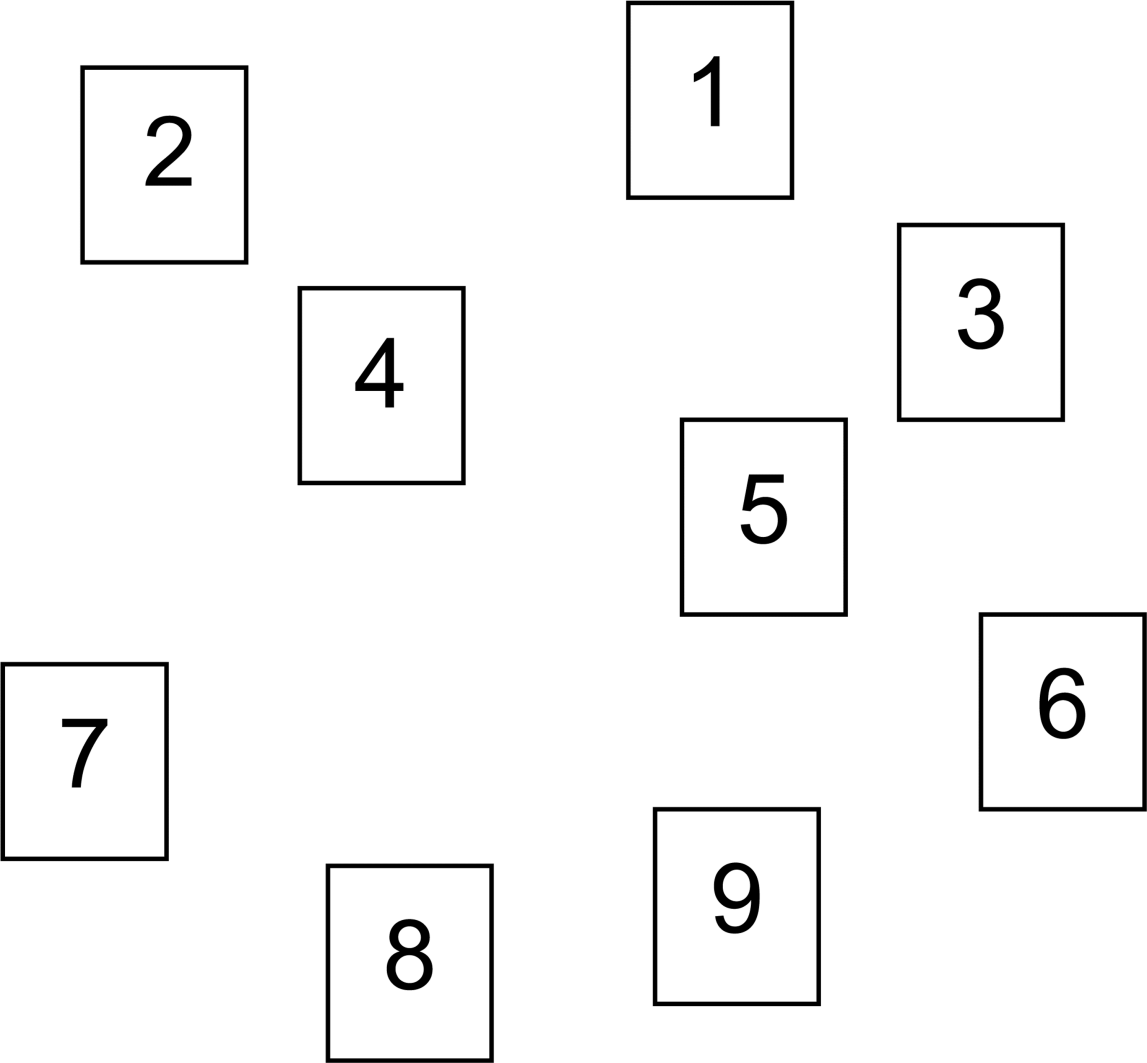
Visuospatial working memory test (CBTT) CBTT, Corsi block tapping task

The CBTT was performed as a visuospatial working memory task (Figure 3) [12]. Nine blocks of squares were presented on paper, and the examinee was asked to point to each square one after the other. Participants were instructed to memorize the order and point to the blocks in the same order for one trial. Two sets were performed per trial, and if at least one set was repeated correctly, the pointing sequence was increased to 3, 4, and so on. Pointing was performed with the index finger at a rate of approximately one per second. The number of successful block sequences was used as the visuospatial working memory score.

The SDQ, a child strengths and difficulties questionnaire, was administered to determine the characteristics of children with developmental disabilities [13]. There were four subscales for difficulties and one subscale for strengths. The SDQ consists of 25 questions, five items for each, to be answered by the parents. The difficulties consisted of 20 items from four subscales: emotional problems, conduct problems, hyperactivity/inattention, and peer relationship problems, and the strengths consisted of five items from one subscale: prosocial behavior. Each item is rated on a three-point scale: 0 for “not applicable,” 1 for “fairly applicable,” and 2 for “applicable.” The total score of the four difficulties subscales (emotional problems, conduct problems, hyperactivity/inattention, and peer relations problems) is calculated as the “total difficulties score” (0-40 points). Furthermore, the questionnaire was administered in paper format, with no changes whatsoever to the wording.

Experimental procedures

The Purdue Pegboard and SDQ were administered one week before the study began. The participants were then assigned to two groups according to their Purdue Pegboard scores, age, and gender: a constant practice group and a variable practice group. On the day of the study, an experimental task (target-aiming task) and a visuospatial working memory assessment (CBTT) were administered. The experiment was conducted in a quiet room, and each participant completed the experiment within approximately 10 minutes.

**Figure 4:**
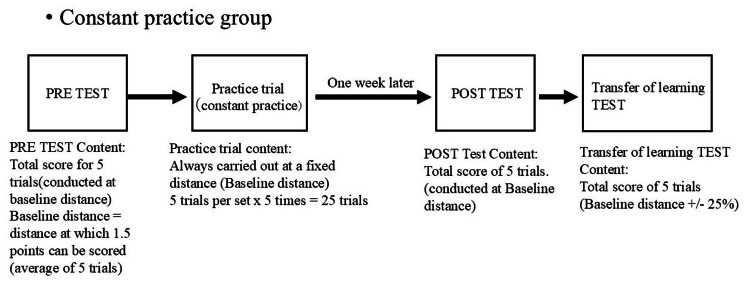
Flowchart for the constant practice group

**Figure 5:**
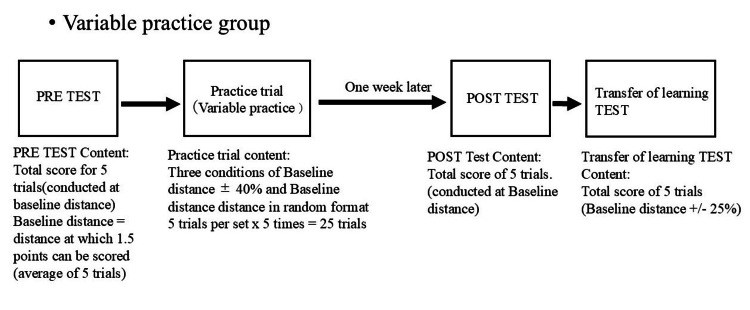
Flowchart for the variable practice group

In the PRE test, the task was to score a total of five trials (the baseline distance at which the average of the five trials could score 1.5 points). The practice trials were always performed at the baseline distance in the constant practice group, with five trials per set × five times = 25 trials (Figure 4). In the variable practice group, the three conditions of baseline distance ±40% and baseline distance were performed in a random format, with five trials per set × five times = 25 trials (Figure 5). One week after the practice trials, the POST test was administered; the POST test tasked the participants with a total score of five trials (baseline distance at which the average of the five trials would score 1.5 points). The transfer test was administered with a total score of five trials (performed at ±25% of the baseline distance). Practice tasks within ±25% of the baseline distance were systematically excluded to prevent contamination of transfer test outcomes through task similarity. Additionally, a standardized break protocol was implemented: five-minute inter-sectional breaks and 30-second inter-task intervals between practice trials.

Statistical analysis

First, normality was confirmed by the Shapiro-Wilk test, followed by a t-test with no correspondence for the comparison of the subjects’ backgrounds between groups. Then, a repeated measures two-way ANOVA was conducted to examine the effects of practice condition (constant practice, variable practice) and time (pre-test, five practice trials, and post-test) on target-taking task scores. The two within-subject factors were (1) practice condition with two levels (constant and variable) and (2) time with seven levels (pre-test, practice trials 1 to 5, and post-test). In addition, the percentage change in target-reward task scores from pre-test to post-test was calculated for each practice condition and compared between groups using an independent t-test. Furthermore, the percentage change in scores during the practice trials relative to the pre-test score was also calculated to evaluate the extent of performance improvement or decline throughout the practice phase. Pearson’s correlation analysis was conducted to investigate the factors associated with the rate of change of scores during practice trials in each practice condition group.

IBM SPSS Statistics for Windows, Version 27.0 (Released 2020; IBM Corp., Armonk, NY, USA) was used for statistical processing, and the statistical significance level was set at 5%.

## Results

The Shapiro-Wilk test indicated no significant deviations from normality (p > 0.05), suggesting that all variables were normally distributed. The basic information of the 34 participants is presented in Table 1. The comparison of age, CBTT, Purdue Pegboard, target-target task, total SDQ score, and components of the SDQ between groups was conducted using unpaired t-tests and χ² tests (Table 1). There were no significant differences between the pre- and post-practice conditions in each practice condition. However, there was a significant difference in the percentage change in scores before and after practice in each practice condition (t-value = 2.976, p = 0.007, d = 1.021) (Table 2). However, there was no significant difference in the transition task scores for each practice condition.

**Table 1:** Group comparisons of underlying characteristics and key indicators (unpaired t-test and χ² test) Values are presented as mean ± SD. SDQ, Strengths and Difficulties Questionnaire

Measure	Constant practice group	Variable practice group	t-values and χ² values	p-values	Effect size (Cohen’s d and phi coefficient)
Sex (number)	Male: 14, Female: 3	Male: 14, Female: 3	0	1	0
Age (years)	9.4 ± 2.3	9.5 ± 2.0	-0.161	0.873	2.137
Working memory score (points)	5.1 ± 1.0	4.8 ± 1.4	0.851	0.401	1.21
Purdue Pegboard score (points)	30.6 ± 5.5	31.1 ± 7.9	-0.202	0.841	6.782
Total SDQ score (points)	20.9 ± 3.8	21.2 ± 7.4	-0.145	0.885	5.896
Emotional symptoms (points)	3.1 ± 2.4	3.2 ± 2.7	-0.203	0.841	2.537
Conduct problems (points)	3.1 ± 1.7	3.3 ± 2.1	-0.357	0.724	1.924
Hyperactivity/inattention (points)	5.8 ± 1.8	5.5 ± 2.8	0.506	0.616	2.372
Peer problems (points)	4.0 ± 1.2	3.6 ± 2.2	0.693	0.494	1.733
Prosocial behavior (points)	4.9 ± 2.0	5.6 ± 2.1	-1.006	0.322	2.045

**Table 2:** Percentage change pre- and post-intervention and transition task scores in the practice condition (unpaired t-test) Values are presented as mean ± SD.

Measure	Constant practice group	Variable practice group	t-values and χ² values	p-values	Effect size (Cohen’s d and phi coefficient)
Transfer task score (points)	2.4 ± 0.8	2.5 ± 0.7	-0.402	0.69	0.738
Rate of change in score during practice trial (%)	17.1 ± 25.0	15.1 ± 44.3	0.168	0.868	35.941
Percentage change in score before and after practice (%)	29.6 ± 61.2	-17.7 ± 23.3	2.976	0.006	46.316

**Figure 6:**
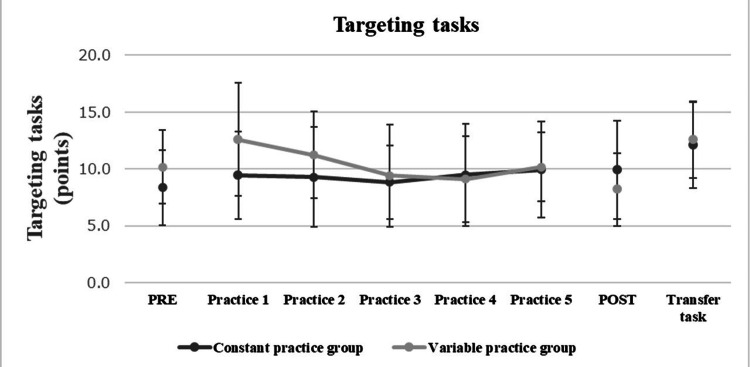
Changes in scores for target-guessing tasks under practice conditions The figure shows changes in target-hitting task scores across the pre-test, practice, post-test, and transfer test for both the constant and variable practice groups. Error bars represent SDs. As shown, the two groups performed similarly during the practice phase and transfer test; however, during the retention test, the variable practice group demonstrated a significant decline in performance compared to the constant practice group.

Figure 6 shows the change in scores on the target-aiming task in the PRE, practice, POST, and transition tests for the constant practice and variable practice groups. First, as supplementary information, each participant performed an average of one to three sets of throws (one set = five throws) during the pre-test to establish a baseline. Repeated measures of two-way ANOVA were performed on the PRE and POST scores obtained in the target-aiming task for each practice condition. The results showed that no significant differences were obtained for the practice condition (F = 0.003, p = 0.956, partial η² = 0.000) and time factor (F = 0.118, p = 0.733, partial η² = 0.004), but significant differences were obtained for the interaction (F = 6.641, p = 0.015, partial η² = 0. 172), (F = 6.641, p = 0.015, partial η² = 0), respectively. The results of the interaction analysis are as follows: We found a significant decrease (1 > 2, p = 0.047) in the pre- and post-target guessing scores when the practice conditions were diverse. Next, a repeated-measures two-way ANOVA was conducted on the scores obtained during the target practice task for each practice condition. The results showed no significant differences in the practice condition (F = 1.405, p = 0.245, partial η² = 0.042), time factor (F = 1.047, p = 0.400, partial η² = 0.126), and interaction (F = 1.375, p = 0.254, partial η² = 0.041). No significant differences were observed between groups. Error bars represent SD. During the practice phase and transition test, the experimental groups were almost identical; however, during the retention test, the variable practice group showed a significant decrease compared with the constant practice group.

Correlation analysis of the rate of change in scores during practice trials (PRE scores/change during practice) and the rate of change in scores before and after practice (PRE scores/change before and after practice) in the constant practice group showed a strong correlation (p = 0.004, r = 0.666) (Table 3). A strong correlation was also found between the rate of change in scores during practice trials and prosocial behavior (p = 0.018, r = 0.564). Correlation analysis of the rate of change in scores during practice trials and the rate of change in scores before and after practice in the variable practice group revealed no correlation (p = 0.145, r = 0.369). No correlation was found between the rate of change in scores during the practice trial and other indices.

**Table 3:** Correlation analysis of the percentage change in scores during practice attempts and the percentage change in scores before and after practice under practice conditions (Pearson’s product-rate correlation coefficient) Values are presented as mean ± SD. SDQ, Strengths and Difficulties Questionnaire

Practice condition	Variable	Correlation coefficient	p-value
Constant practice group	Age (years)	-0.51	0.36
	Working memory score (points)	-0.031	0.907
	Total SDQ score (points)	-0.238	0.358
	Emotional symptoms (points)	-0.078	0.767
	Conduct problems (points)	0.155	0.554
	Hyperactivity/inattention (points)	0.035	0.894
	Peer problems (points)	0.051	0.846
	Prosocial behavior (points)	0.564	0.018
	Percentage change in score before and after practice (%)	0.666	0.004
Variable practice group	Age (years)	0.193	0.459
	Working memory score (points)	0.192	0.461
	Total SDQ score (points)	0.28	0.277
	Emotional symptoms (points)	0.371	0.143
	Conduct problems (points)	0.368	0.146
	Hyperactivity/inattention (points)	0.109	0.677
	Peer problems (points)	-0.003	0.99
	Prosocial behavior (points)	0.001	0.998
	Percentage change in score before and after practice (%)	0.369	0.145

## Discussion

The purpose of this study was to investigate the effects of different practice conditions, such as constant practice and variable practice, on motor performance and its changes in children with ASD with motor coordination disorder and to examine practice methods that promote motor learning in children with ASD with motor coordination disorder.

First, the present study compared the groups in each practice condition and found no significant differences in gender, age, CBTT, Purdue Pegboard, total scores on the SDQ, or components of the SDQ. Each effect size (Cohen’s d) was also small [14], suggesting that it was difficult to find clear differences in gender, age, CBTT, Purdue Pegboard, total SDQ scores, and SDQ components by practice condition. These results suggest that, at least in the present practice condition, the direct effects of gender, age, CBTT, Purdue Pegboard, total SDQ scores, and SDQ components on the target-reference task were not pronounced.

Subsequently, significant differences were found in the percent change in scores before and after practice in each practice condition. This result suggests that the practice condition may have affected motor learning in children with ASD with motor coordination disorder. In particular, the effect size (Cohen’s d) was as large as 1.021 [14], indicating that there was a significant difference in the rate of change in scores before and after practice between the constant practice group and the variable practice group. The significant difference between the pre- and post-practice score change rates may be because the variable practice and constant practice conditions may have contributed to the improvement of the participant's performance in different ways. In a detailed analysis, a significant difference in interaction was found in the repeated measures two-way ANOVA, and a significant decrease was found in the target shooting task after practice compared to before practice when the practice condition was variable practice. These results suggest that variable practice in particular may not be suitable for motor learning in children with ASD with motor coordination disorder.

In general, it has been reported that, as a practice condition with high motor learning effects in typically developing children, variable practice, in which tasks are practiced with variability, has a more positive effect on motor learning effects than constant practice, in which tasks are practiced repeatedly under constant conditions [9]. This is because practicing movements that vary in duration and force but have the same order of elements and basic temporal structure, as in variable practice, leads to the formation of motor schema [9]. However, such motor learning theory is not necessarily applicable to individuals who cannot form motor schemas. As mentioned above, it has been reported that in people with AD, motor learning was more effective in constant practice and rather less effective in variable practice [10]. This may be due to the difficulty in parallel processing of multiple task information due to a decrease in visuospatial working memory, and the inability to form motor schema appropriately [9]. Therefore, we expected a similar cause in children with ASD with motor coordination disorder, who were the subjects of this study. However, since no significant difference in visuospatial working memory was found between the constant practice group and the variable practice group in this study, it is necessary to clarify in future research what causes variable practice to be unsuitable for motor learning in children with ASD with motor coordination disorder. In addition, since all of the subjects in this study were able to perform the throwing task before the experiment, it is unlikely that the failure to learn when the practice conditions were varied was due to problems in understanding the instructions or paying attention to the task. In light of the above, it is necessary in the future to examine exercise programs based on practice conditions for children with ASD with motor coordination disorder. Because of the difficulty in forming motor schema, it is predicted that constant practice was more suitable for the children with ASD than variable practice. In support of these predictions, Figure 6 shows the change in scores for the guessing task under the practice condition. This can be attributed to the fact that the schema theory of rehabilitation schema was not formed well. These performance curves suggest that ASD children with motor coordination disorder, such as the subject in this study, are unable to cope with changes in tasks when variable practice is applied in the early stages of practice.

On the other hand, there were no significant differences in transfer task scores between practice conditions. The fact that no effect was observed between groups suggests that the practice condition may not have had a significant effect on the improvement of transfer ability. It is known that a certain degree of similarity between the original practice condition and the transition task is generally required to observe the effect of the transition task, and in this study, the relationship between the practice condition and the transition task was fully considered, but no significant differences in the transition task were observed between the practice conditions. This result suggests that differences in practice conditions, such as constant practice and variable practice, may not have a significant effect on the ability to transfer in children with ASD with motor coordination disorder. In general, it has been reported that children with typical development have better retention and transfer ability in the variable practice group than in the constant practice group [15-17], but the results of the present study were different. This transfer ability means that it is important to improve transfer ability when the goal is to apply the skills to competitive sports or daily life, but the transfer is not necessarily necessary when the goal is to improve a specific skill. Therefore, changes in scores before and after practice may be more important for enhancing the specific skill of throwing a ball, as in this study.

Next, a repeated measure two-way ANOVA on scores during target practice, but not before and after practice, showed no significant differences in practice condition, time factor, or interaction effects. This result suggests that neither the practice condition nor time had a significant effect on the change in scores during practice. In other words, the results suggest that the practice condition makes no difference in performance during practice and that it is in the post-practice phase that the differences in practice condition become more pronounced.

We then examined the relationship between the rate of change in scores before and after practice and the rate of change in scores during practice trials for the constant practice group and the variable practice group, respectively. In the constant practice group, there was a correlation between the rate of change in scores before and after practice and the rate of change in scores during the practice trial. On the other hand, in the variable practice group, there was no significant difference between the rate of change in scores before and after practice and the rate of change in scores during the practice trial. In other words, rather than concluding that the constant practice group showed a generally higher rate of improvement from pre- to post-test, the findings suggest that, within this group, a greater rate of improvement during the practice trials may have contributed to the observed motor learning gains. However, this interpretation should be viewed with caution, as it may not be directly generalizable beyond the specific context and characteristics of the participants in this study. Therefore, it is necessary to examine what factors are related to the rate of change in scores during practice trials.

Next, we conducted a correlation analysis to examine how the rate of change in scores during the practice trials in the constant practice group related to age and each component of the SDQ, including emotional problems, conduct problems, hyperactivity/inattention, peer relationship problems, and prosocial behavior. This analysis aimed to explore the characteristics of children who demonstrated a higher rate of score change during practice. As a result, a significant positive correlation was found only between the rate of score change during practice and prosocial behavior in the constant practice group. This finding suggests that children who showed greater improvement during practice may tend to exhibit stronger prosocial behaviors. A previous review study on children with DCD - a population distinct from the children with ASD in the present study - reported that motor difficulties often limit participation in social activities, leading to isolation, anxiety, and emotional or social challenges for both the children and their families [18]. While that study emphasized the negative impact of coordination difficulties on social functioning, our current findings highlight the potential positive role of prosocial behavior in supporting motor learning under structured, repetitive practice conditions. Therefore, rather than assuming that children with ASD and coordination difficulties universally exhibit low prosocial behavior, it is important to consider the possibility that some children possess and demonstrate positive social traits that may facilitate learning. Further studies are needed to clarify the nature and direction of the relationship between prosocial behavior and motor learning in this population.

A limitation of this study is that the age range of the subjects in this study was relatively wide, ranging from six to 15 years old. The reason for the target age range of the present study was that coordination motor disorder tends to be a problem during the school age [19], and therefore, we recruited subjects in the school age range. Since the school age differs from country to country and culture to culture, this study referred to the definition of school age in the Japanese Basic Education Law [20]. According to the Basic Education Law, the definition of school age in Japan is from six to 15 years old [20], and this definition was adopted. In this study, age was adjusted for homogeneity when dividing the children into groups, and subsequent comparisons between groups showed no significant differences in age. However, many reports have shown that motor skills [21] and visuospatial working memory [22] also differ depending on the developmental stage, so it may be necessary to narrow the age range or increase the sample size and conduct age-specific analyses in the future. The sample size for this study was 34 persons. The preliminary sample size required was 34 persons (calculated as 1-β = 20% and α = 5% in the effect size), which was calculated based on the previous study, and the target sample size was secured. The effect size of the t-test between groups in this study (d value of 1.021) was at a high level, and the repeated measures two-way ANOVA interaction (η² of 0.172, corresponding to d = 0.7) was at a moderate level, which was almost consistent with the expectations of the study design. Furthermore, in studies similar to the present study on children with developmental disabilities, most of them had sample sizes similar to those studied here [23,24]. However, there are still few studies in the field of children with developmental disabilities that clarify the effects of different practice conditions on motor learning, as in the present study. Therefore, further studies with a larger sample size and more precise estimation of the effect size will be necessary in the future. In addition, a detailed intelligence test was not conducted on the children in this study. We recruited subjects with an IQ of 70 or higher, as information in their preliminary medical records. However, because intellectual function is related to the severity of motor impairment [25], a detailed examination of intellectual function should have been conducted. In addition, standardized questionnaires (e.g., Social Responsiveness Scale-Second Edition) should have been used to assess the severity of ASD and individual symptoms, in addition to the diagnosis of ASD from the physician, although the subjects in this study were children with ASD. Because the distribution of ASD symptoms is continuous regardless of diagnosis, suggesting that specific ASD symptoms may influence motor impairments [26], it is necessary to assess whether there is a correlation between participants’ ASD characteristics and the study results. In the present study, the SDQ was used to understand the characteristics of children with ASD; some studies have examined the SDQ for developmental disability characteristics [27,28]. Among these studies, Saito et al. [27] showed that high autistic traits in young children predicted the risk of mental health problems such as “friendship problems” and “emotional problems” after schooling. Moriwaki and Kamio [28] also reported from national data that children with high autistic traits are more likely to have mental health problems. In other words, the SDQ is likely to be suitable for understanding autistic characteristics. However, future research should utilize standardized questionnaires specific to ASD and more accurate testing. Finally, as this study was the first to investigate the effects of practice conditions on motor learning in children with ASD and co-occurring coordination disorders, it was not possible to determine the number of practice sessions based on previous research. The lack of significant improvement in task scores from pre- to post-test may be attributed to both the characteristics of the participants and an insufficient number of practice sessions. Therefore, future studies should consider implementing a greater number of practice sessions to more effectively assess motor learning outcomes in this population.

## Conclusions

This study compared the effects of constant practice versus variable practice on motor learning in children with ASD with co-occurring motor impairments. Thirty-four children underwent assessments of fine/gross motor skills, visuospatial working memory, and developmental disabilities. Performance changes in a target task were analyzed before, during, and after practice. Results showed no significant main effects of practice type or time alone, but a significant interaction effect. Variable practice led to reduced post-test performance, while constant practice demonstrated stronger correlations between practice improvements and overall performance changes. Prosocial behavior was positively associated with motor learning gains in the constant practice group. The findings suggest structured constant practice enhances motor learning more effectively in children with ASD and highlight the potential benefits of integrating motor training with social skill development.
